# Neuroplasticity beyond Sounds: Neural Adaptations Following Long-Term Musical Aesthetic Experiences

**DOI:** 10.3390/brainsci5010069

**Published:** 2015-03-23

**Authors:** Mark Reybrouck, Elvira Brattico

**Affiliations:** 1 Section of Musicology, Faculty of Arts, KU Leuven—University of Leuven, Blijde-Inkomststraat 21, P.O. Box 3313, 3000 Leuven, Belgium; 2 Faculty of Psychology and Educational Sciences, Center for Instructional Psychology and Technology, KU Leuven—University of Leuven, Dekenstraat 2, P.O. Box 3773, 3000 Leuven, Belgium; 3 Helsinki Collegium for Advanced Studies, University of Helsinki, Fabianinkatu 24, P.O. Box 4, 00014 Helsinki, Finland; 4 Cognitive Brain Research Unit, Institute of Behavioural Sciences, Siltavuorenpenger 1 B, P.O. Box 9, 00014 Helsinki, Finland

**Keywords:** adaptation, neuroplasticity, music, aesthetic experience, emotion

## Abstract

Capitalizing from neuroscience knowledge on how individuals are affected by the sound environment, we propose to adopt a cybernetic and ecological point of view on the musical aesthetic experience, which includes subprocesses, such as feature extraction and integration, early affective reactions and motor actions, style mastering and conceptualization, emotion and proprioception, evaluation and preference. In this perspective, the role of the listener/composer/performer is seen as that of an active “agent” coping in highly individual ways with the sounds. The findings concerning the neural adaptations in musicians, following long-term exposure to music, are then reviewed by keeping in mind the distinct subprocesses of a musical aesthetic experience. We conclude that these neural adaptations can be conceived of as the immediate and lifelong interactions with multisensorial stimuli (having a predominant auditory component), which result in lasting changes of the internal state of the “agent”. In a continuous loop, these changes affect, in turn, the subprocesses involved in a musical aesthetic experience, towards the final goal of achieving better perceptual, motor and proprioceptive responses to the immediate demands of the sounding environment. The resulting neural adaptations in musicians closely depend on the duration of the interactions, the starting age, the involvement of attention, the amount of motor practice and the musical genre played.

## 1. Introduction

Since the advent of neuroimaging, in the early 2000s, music has been studied as a biological faculty by means of controlled experiments, allowing quantitative observations on healthy brains in perceiving or performing tasks (for a comprehensive overview of the field, see [1]). A dominant model put forward by Isabelle Peretz on the basis of brain-lesion evidence postulated the fragmentation of music into modules, which were deemed to be automatic, encapsulated and independent of each other [2]. Subsequently, Anirrudh Patel challenged this view by proposing non-modular resources shared between language and music (the “shared syntactic integration resource hypothesis” or SSIRH [3]). Stefan Koelsch [4] noticed the recruitment of the brain mechanisms and structures (in the pars opercularis of the inferior frontal gyrus) for the processing of harmony rule violations, which are homologous to those needed for processing rules in language syntax, although predominant in the right-hemisphere (as opposed to brain structures for language syntax, mainly located in the left hemisphere). A more bottom-up hypothesis about the brain specialization for processing language sounds as opposed to musical sounds has been proposed by Robert Zatorre and colleagues [5].

The underlying assumption in the field of cognitive neuroscience was a conception of music as non-verbal auditory communication, strictly related to language and comparable to or distinguishable from it. Within this framework, a musical experience is typically studied by designing experiments where sounds are manipulated one feature at a time and the subjects’ behavioral responses to those feature manipulations being correlated with corresponding brain signals. The increasing interest in understanding subjective emotional processes in music, on the other hand, has added a new stance moving towards the objective measurement of pleasure and enjoyment of music [6], and attempts are being made to explain how predictions of incoming events, processed cognitively according to the rules of a musical culture, give rise to emotional responses [7,8]. Up to now, however, only a few scholars have attempted to study with neuroscientific methods the whole experience of music in the ecological setting of an everyday experience.

Recently, Brattico *et al.* [9] envisioned the musical experience as an instance of an aesthetic process, to be studied in relation to other art forms, rather than as a cognitive domain. Hence, the importance of considering the aesthetic attitude or “stance” [10], namely the intentional contemplative approach to an object, which is perceived, conceptualized and evaluated as detached from its utilitarian functions [11,12,13]. In their article, they review the available neurophysiological and neuroimaging evidence to delineate the main brain structures and mechanisms involved in the distinct stages of an aesthetic musical experience. As such, they outline a chronometric approach that views the musical aesthetic experience as dynamically evolving in time with subprocesses, such as feature extraction and integration, early affective reactions and motor actions, cognitive style mastering, emotion and proprioception, evaluation and preference. This and other frameworks developed by music psychologists [14] point at the importance of the listener and the context in feedback loops that constantly modify the quality of the experience. Hence, according to these frameworks, the long duration, high emotional intensity and focused attention of a musical experience in music academy students and professional musicians are supposed to substantially modify its component structure, such as the neural mechanisms related to feature encoding integration, or the brain structures important for emotion and evaluation, or even the chronometric succession of the aesthetic processing stages. Possibly, neural modifications might occur from the passive exposure to a specific musical culture, as opposed to another one; however, since very little research is dedicated to this topic, we opt to discuss findings related to music experts, such as professional musicians or students from a music academy.

**Figure 1:**
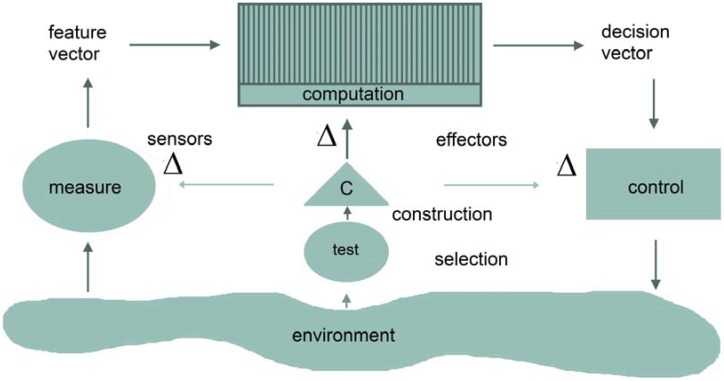
Schematic depiction of a structurally-adaptive device that can alter its sensors, effectors and computational part. The symbol Δ denotes the adaptation or change at one or more of the major moments of the system, and the letter C stands for construction (adapted from Cariani [26], with permission).

**Figure 2:**
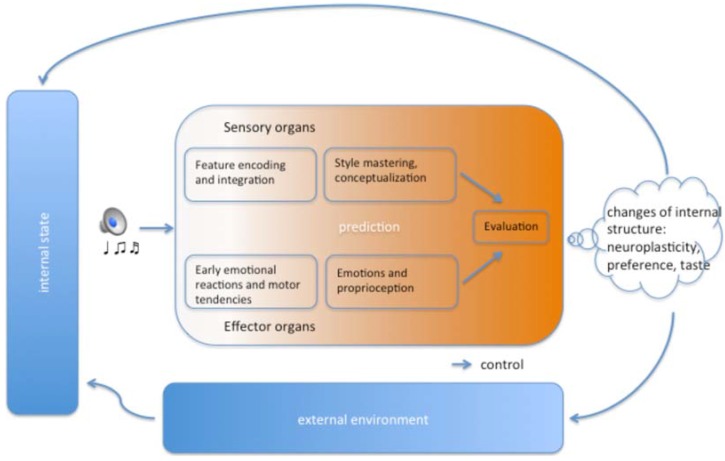
Schematic illustration of the subprocesses of a musical aesthetic experience (simplified and adapted from Brattico *et al.* [9]) and how repeated exposure modifies the internal structure of the music agent, consequently changing his or her external environment and internal state in a continuous loop.

In this contribution, we focus on how the prolonged exposure to an aesthetic musical experience in musicians might alter each stage of the process. For doing so, we integrate the previous model by Brattico *et al.* with concepts from “second-order cybernetics” [15,16,17,18,19,20,21,22], which means that we propose to view the individual exposed to a musical aesthetic experience not as “reactive”, but as an “adaptive” device, with contingent changes of its internal state to respond to the immediate demands of the sounding environment, which in the long-term may lead to changes of its internal structure (for a schematic representation of how these changes are implemented, see Figure 1 and Figure 2 ([22,23,24,25], and for a general discussion of adaptive devices, see [26,27]). In other words, the role of the music listener, performer or composer is that of an active “agent” coping in highly individual ways with the sounds. Hence, “music agent” is seen here as a broad category, somewhat analogous to the concept of “music user” that was proposed by Laske [28] to encompass all subjects that experience music in some way. The environmental context and its related dynamics become central, therefore, when considering an *aesthetic experience*, not only because they allow (or not) a dedicated voluntary attention state for contemplation of a piece of art, but also since they impact on putative motor actions, described as “aesthetic affordances” or “structural action invitations” [10], such as walking around an installation, approaching a sculpture or closing the eyes to focus on the music.

From the perspective of second-order cybernetics, the plastic brain of “music agents” can be viewed as a structurally-adaptive device [26,29], which relates sensors and effectors through mental computations and which can modify its internal structure, as well (see Figure 1). It can be schematized in terms of input-output relationships with the functional equivalents of measuring and controlling being expressed as feature and decision vectors. In addition to these “sensor-effector loops”, however, the device has a “feedback-to-structure loop”, as well, that evaluates or tests performance and alters sensing, computation and effector actions in order to improve measured performance. Evaluations, in this view, are able to trigger a change in the system’s structure rather than its state [30], enabling new functional states and operations to arise. In neuroplastic terms, this means that the process of neural adaptability or plasticity, mainly consisting of the reorganization of the synaptic contacts between neurons, is triggered by intense and challenging environmental demands, which are able to modify the design of the brain circuitries, not only as the outcome of early experiences, but throughout the whole life span. The neural circuits, in other words, are not totally pre-given and wired-in, but are repeatedly pliable and modifiable by continued sensory experience (see [31,32,33,34,35,36,37] and [38] for musical applications).

Starting from this operational approach, we propose a neuroscience-centered perspective on the musical experience relying on a cybernetic and ecological approach that considers music agents as “organisms” that cope with their environment, calling forth a broader conception of skill acquisition and plasticity that broadens the research from mere action-oriented research to the level of perception and sense-making, as well. The final goal is to view a musical experience as an active process of sense-making, where the individual characteristics of each music agent (and even the actual mental state in a particular moment of life) might largely decide what is considered as music and what is not, what kind of music is chosen to listen to or to play and how that music is subjectively experienced.

By adopting the above delineated concepts and frameworks, we programmatically review the literature on neuroplasticity in prolonged musical engagements, by including all of the different subprocesses constituting a musical experience under an “aesthetic stance” (as conceived of by Brincker [10]). Only some of these subprocesses have been studied from the perspective of long-term neuroplastic changes, and no attempt has been made to illustrate the putative changes in the chronometric succession of aesthetic subprocesses. Hence, where studies are not yet conducted, hypotheses for stimulating new research will be put forward. In what follows, we summarize training-related neural adaptations in all relevant subprocesses of a musical aesthetic experience, following a framework simplified from Brattico *et al.* [9]. We conclude by proposing a schematic model of how musical exposure might interact with these subprocesses (Figure 2).

## 2. Feature Encoding and Integration

Basic music feature processing (such as pitch, timbre, contour and duration of sounds), though dependent on universal dispositional abilities [39,40], may vary considerably across individuals. Relevant to the present paper, this individual variation is dependent on the amount of repeated exposure to a definite environment, which in the music domain corresponds to a specific musical culture and/or instrumental training. As such, there is a possible tension between wired-in circuitry for perceptual information pick up against the learned mechanisms for information processing and sense-making that are the outcome of immersion in a culture [41]. The former involves a lot of reactive behavior that points in the direction of direct processing beyond conscious and deliberate control, such as automatic processing of pitch variations in the auditory cortex (as indexed by the mismatch negativity (MMN) of the event related potential (ERP); for a review, see [42]).

Relatively little is known about neuroplastic adaptations related to basic musical feature processing after mere exposure to a specific musical culture as compared to another one (e.g., [43]). Nevertheless, studies on the consequences of instrumental training restricted to one musical culture, on the initial subprocesses of a musical experience, involving basic aspects of music perception, are abundant. These plastic changes are numerous and well documented [44,45,46,47,48,49,50,51,52,53,54,55,56,57] and have shown convincingly that long-term musical practice can lead to both anatomical (or structural) and physiological (or functional) changes, which, in turn, affect basic aspects of music perception. For instance, neurophysiological studies have shown that music experts are able to discriminate tiny changes in musical sound features (such as pitch, duration, timbre, dynamics, *etc.*), thanks to their more accurate neural representations in the auditory cortex, particularly in the right hemisphere [58,59,60]. These physiological adaptations are accompanied by anatomical changes (see [61] for an overview), e.g., the grey matter of Heschl’s gyrus, where the primary auditory cortex is located, which is enlarged by long-term exposure to music, such as happens when learning to play a musical instrument in a conservatory [62]. Related to this, there is direct evidence about the causal effects of music training on anatomo-physiology and cognitive functions in childhood: structural (with a linear positive correlation between the magnitude of neural adaptations in the brain and the years of musical training [51,59,63,64]), functional [65] and transfer effects from musical to other cognitive functions [66,67].

The auditory cortex is also altered by the content of a specific music listening experience, namely the possession of *absolute pitch*. The peculiar ways of listening to sounds by musicians with absolute pitch—the rare ability to immediately and effortlessly recognize or produce the pitch of a tone without using an external reference [68,69,70,71]—is reflected in the larger volume of the planum temporale in the left hemisphere (a posterior region of the superior temporal gyrus, where the non-primary auditory cortex is located) or the reduced size of the right planum temporale as compared to musicians not possessing absolute pitch [72,73,74,75]. The surface area of that structure has long been taken as a structural marker for left hemisphere language dominance in right-handers and is found to be asymmetric in normal right-handed samples with a greater leftward bias [73]. The functional significance of the left-sided asymmetry of the planum temporale in possessors of absolute pitch can thus be seen in the context of their ability to assign any pitch to a verbally-labelled pitch class, which, in turn, influences the recognition memory for pitch [44,74,76,77]. Further investigations evidenced that even the intra-hemispheric connections between auditory regions are altered in absolute pitch possessors. By means of diffusion tensor imaging (DTI) of the white matter tracts, Loui *et al.* [78] obtained increased structural connectivity between the superior and middle temporal gyrus, especially in the left hemisphere in absolute pitch possessors.

These basic processing abilities for feature encoding and integration, which were reviewed above, are only the first stages of music processing. In the following section, we proceed to review findings related to the next subprocesses in a musical aesthetic experience, namely early emotional reactions and motor tendencies, and how they are modified after prolonged musical practice in musicians as compared to non-musicians.

## 3. Early Emotional Reactions and Motor Tendencies/Actions

### 3.1. Early Emotional Reactions and Involuntary Motor Tendencies 

Music is a powerful tool for expressing and inducing emotions [79,80,81]. Musical emotions are of many kinds and include the automatic reactions to sounds, the conscious categorization of sounds, e.g., as happy or sad, or the intentional enjoyment while listening to a favorite piece. In a chronometric succession, the first affective responses to musical sounds have been termed early emotional reactions [9] or brainstem reflexes [79]: they occur with rapid onset, through automatic appraisal and with involuntary changes in physiological and behavioral responses [82]. Such automatic or reflex-like emotional reactions to, e.g., loud or dissonant sounds involve evolutionarily older structures of the brain, such as the hypothalamus, brainstem and limbic system (for a review, see [9]). They point in the direction of direct processing beyond conscious and deliberate control and involve a lot of biological regulation that engages less developed structures of the brain that are specialized for their processing [11,79,82,83,84,85,86,87,88,89,90,91,92].

There is increasing evidence, further, that musical emotions are quickly and easily perceived by members of the same culture (for a critical review, see [83]) and that emotional judgments exhibit a high degree of consistency, suggesting that perception of emotions in music is natural and effortless for the large majority of listeners [82]. This is considered valid also for early emotional reactions to sounds in general. Moreover, early emotional reactions are often accompanied by involuntary motor tendencies, including bodily changes related to the response of the autonomic nervous system (mainly the sympathetic one) to the sound events and are in many cases, such as with basic emotions, constitutive of what identifies an emotion [93]. Among the most studied involuntary bodily reactions are the chills or frissons, namely shivers down the spine, or goose-bump skin, sometimes accompanied even by crying, in response to listening to very pleasurable, familiar music (see [94,95,96,97] for an overview). A behavioral and physiological study by Grewe *et al.* [98], however, did not find any difference in the frequency of the occurrence and nature of the chill responses in musicians as compared to non-musicians while they listened to pieces by Bach, Mozart and Puccini; on the other hand, familiarity with the music increased the frequency of chills.

Another early reaction to sound, which is associated with bodily changes, is related to sensory dissonance, namely the unpleasant sensation when listening to sound combinations, which include slow amplitude modulations (or beats), as the result of slight frequency differences between the constituting tones. Dissonance is considered as a perceptual universal (e.g., [99]). Recent findings, however, demonstrate that it can substantially vary depending on the musical experience and even on individual anatomical peculiarities of the auditory pathway. One study showed that sensory dissonance induced more unpleasant feelings and stronger physiological reactions, measured with skin conductance and electromyography, in individuals with long-term musical training than in those with a lower amount of training [100]. Moreover, the anatomy of the inferior colliculus, a brainstem relay structure of the auditory pathway, explains variations in dissonance perception among individuals [92]. Whether and how much this anatomical feature exists irrespectively of musical training and whether it relates to the possession of inborn musical abilities has yet to be understood.

### 3.2. Voluntary Motor Tendencies/Actions

Other motor tendencies/actions are controlled by the central nervous system and require the conscious will of the subject, at least for initiating the movement. These motor actions or tendencies can be distinguished in those aiming at a response to listening to music and those that are required to produce sounds as in motor practice with an instrument. It is well known that the latter type of motor actions in prolonged instrumental practice can induce long-term neural adaptations. The voluntary motor actions needed to play a musical instrument, involving limb and/or facial movements, which may be repeated for several hours per day, continuing for several years and often starting at an early age (typically around school age, but sometimes even earlier), can modify the volume of the grey matter of the primary motor cortex [101] along with other auditory and visuospatial areas also involved during musical practice [33,102]. Cortical areas of the frontal lobe of the encephalon, the basal ganglia and the cerebellum play a particular role in the temporal control of sequential motor tasks and their integration in bilateral motor behavior (as in keyboard and string players) and, hence, are also significantly shaped by musical practice [73,102].

Furthermore, training-induced changes in white matter brain structure involved in motor practice have been found, supporting the hypothesis that musical training can induce changes in cross-hemispheric connections, such as the corpus callosum, but differences in intra-hemispheric fibers have been found, as well. The corpus callosum, as a typical example of inter-hemispheric connections, is shaped by musical practice. It has been proposed that environmental stimuli, such as intense bimanual motor training of musicians, especially early in life, might affect the determination of callosal fiber composition and size. As such, the corpus callosum has been found to be larger in performing musicians who started their musical training before the age of seven [73,103]. The intra-hemispheric connections, on the other hand, embrace structures as the arcuate fasciculus, superior longitudinal fasciculus, inferior longitudinal fasciculus and uncinate fasciculus and fibers related to motor function, such as the corticospinal tracts and cerebellar peduncles, but these findings have not always been replicated, and findings regarding the internal capsule and corticospinal tracts appear to be contradictory [104]. Changes in the corticospinal tract are not less important, but they did not initiate a consistent body of research up to now [105,106,107].

The repeated motor actions needed for musical practice, further, can also change the neural processing of music, by involving even motor regions in the middle and superior frontal gyri during mere listening, not requiring any motor action [108]. In a very recent study [109], it was found that the bimanual symmetric coordination in musicians and particularly in keyboard players increased the volume of the anatomical structure of the corpus callosum and rendered more symmetric the neural responses of the temporoparietal and inferior frontal areas during music listening. These findings show a coupling of perception and action (sometimes called the motor theory of perception [110,111,112], as evidenced by modern research on the relationships between perception, imagery and motor preparation [113,114,115,116,117,118,119,120,121,122,123], complying with findings on mirror and canonical neurons. The latter can be considered as sets of neurons, which discharge when an individual simply observes an object without performing any movement [115,124,125,126]. They play a pivotal role in the process of transforming visual information of objects or movements into appropriate motor acts, because of the congruence between the codified motor features and the perceived visual properties. The “embodied paradigm” in music theory, as coined by Leman and Godøy [127,128], has incorporated this neuroscientific knowledge of the mirror neuron system by stipulating the importance of understanding musical sounds as inseparable from body movement and, more precisely, to understand any sound as inseparable from the action trajectory required to produce it [129]. Based on these views, motor imagery can be conceived of as a dynamic state during which a subject mentally simulates a given action [130]. Such phenomenal experience of performing a given action without actual manifestation of this action corresponds to the so-called internal imagery (or first person perspective) of sport psychologists and is considered as “ideomotor” rather than “sensorimotor” activity [118]. The transition from overt action to internalized forms of action, however, does not imply the abandoning of the sensori-motor control systems that link the sensors to the central nervous system and the effectors (the muscles). It only cuts off the actual manifestation of the output or effector side of the control system. Additional arguments come from action observance with expert dancers showing that their mirror system integrates observed actions of others with their personal motor repertoire, which suggests that the human brain understands actions by motor simulation [131]. Perception thus involves the same neural substrates as action (the supplementary motor area), and the same holds true for imagined action (see [132,133,134], and for a discussion of the mirror neuron theory in the music domain, see [135]).

## 4. Style Mastering and Conceptualization

In order to reach an aesthetic response to music, some cognitive processing is implied, such as the implicit or explicit understanding of the music’s formal structure. Evidence for this claim has been provided by the phenomenon of congenital amusia, where the learning of music conventions, like harmonic structures, is not possible, due to reduced connectivity between frontotemporal brain structures [136,137,138]. One has to take into account that music, like language, is a complex signal with elements organized according to a culturally determined hierarchy of importance (such as tonality, harmony and meter). The exact nature of these hierarchies, however, is still under debate, with traditional theories posing top-down rules and novel ones proposing dynamic attending as a flexible, bottom-up interaction between external input and internal attending oscillatory processes [139]. Processing these and other hierarchical rules requires the use of higher cognitive functions, including memory and attention, that follow the neural processing of basic sound features, but that, in our model, precede the conscious perception and induction of emotions [9]. The detection of an unexpected sound or a chord, violating or deviating from the conventions of tonal harmony, requires the integration of auditory events over time using working memory processes, the hierarchical organization of those events based on schematic knowledge stored in long-term memory, and hence, the recruitment of attentional resources and prefrontal brain structures [140,141]. This recruitment is definitely intensified after prolonged musical training to a particular musical culture, such as Western tonal music, resulting in accurate neural representations of the hierarchical sound relations in musicians as compared to non-musicians [142]. These findings have been obtained by measuring a neurophysiological brain response, termed early right anterior negativity (ERAN), originating bilaterally, but with right-hemispheric predominance, from the pars opercularis of the inferior frontal gyrus (corresponding to Broca’s area in the left hemisphere). It should be noted also that the multicultural musical experiences of folk musicians seem to weaken the neural representation of Western harmony chord hierarchy, as indexed by the ERAN to chords in a different position within a seven-chord cadence [143].

The hierarchical processing of rules and conventions of sound successions, which plays a central role in the building of an aesthetic emotional response, is linked also with semantic memory, which, in turn, is related to long-term concepts and structures that characterize a particular musical style. As such, it can be stated that familiarity with a musical style determines the formation of online schematic expectations when listening to it, with the anterior temporal cortex of the left hemisphere appearing to be particularly involved in semantic memory retrieval for music regardless of contextual information [144]. Even if the time course of musical semantic memory is not yet fully understood, presumably, as in speech, the processing of semantic features occurs early in the aesthetic experience, largely involving frontal regions of the brain (e.g., [145,146]), which is related, in turn, to a wide range of higher cognitive functions, including self-monitoring, short-term working memory and self-reflection of one’s own emotional states [147].

Various aspects of cognition thus seem to influence music perception and recognition to a much greater extent than motor abilities alone [67]. These aspects include attention [61,148], executive functions [66,149,150], verbal intelligence [66], fluid intelligence [151] and working memory [151,152]. Some scholars even proposed that the neural adaptations induced by musical training depend on the changes in executive functions that derive from musical training, namely inhibitory control, verbal working memory and attention [149]. Indeed, supported by correlations between auditory working memory and attention and auditory brainstem response properties, Strait and colleagues [148] suggested that musicians’ perceptual and neural adaptations towards superior neural efficiency of basic auditory processes are driven in a top-down manner by strengthened cognitive abilities with musical training.

## 5. Emotion and Proprioception

Subsequent to the stage of conceptualization, music agents should be able to perceive, recognize and classify musical emotions, transforming them into conscious feelings that require cognitive and linguistic processes (see Figure 2). In a recent fMRI study [153], brain structures related to cognitive control, working memory and attention in the parieto-occipital and lateral prefrontal cortex were predominantly recruited during the explicit classification of sad, angry and happy emotions, whereas limbic areas, such as the amygdala, were more active during implicit processing of emotional music clips (when listeners concentrated on classifying the clips according to the musical instruments heard). Hence, one can hypothesize about emotional responses that are at least partially modulated by musical expertise, particularly when those responses are governed by the psychological mechanisms of expectation [13] or anticipation [7].

A meta-analysis of 24 neuroimaging studies on musical emotions in subjects undifferentiated according to their musical background showed consistent activity in limbic and paralimbic brain structures, namely the amygdala, the nucleus accumbens, the hypothalamus, the hippocampus, the insula, the cingulate cortex and the orbitofrontal cortex [154]. Some of these regional activations seem to be modulated by musical expertise. For instance, musicians showed enhanced responses of the right ventral striatum and also the anterior cingulate cortex during listening to expressive music (a prelude by Chopin played by a pianist) as compared to non-musicians in an fMRI study [155]. These results, showing increased limbic system reactivity to musical emotions in musicians partly contradict previous behavioral studies that used implicit tasks (such as tasks using emotional stimuli, but diverting the attention away from their emotional content, by asking to classify the number of instruments or the gender of a vocal sound, and so on) and did not find any advantage of musical expertise for musical emotion classification [89,156]. In turn, Chapin *et al.*’s results are in line with other behavioural results using explicit emotion classification task, evidencing musician’s superiority for recognizing emotions in speech prosody (or pitch cues in speech) as compared to musically untrained individuals [157,158]. Furthermore, a behavioural and physiological study [159], utilizing as stimulation sad, happy and scary unfamiliar tunes played by a violinist in an expressive or mechanical way, confirmed that musicians gave higher ratings of emotional intensity for the expressive excerpts as compared to non-musicians. However, only the physiological measure of the corrugator face muscle (as measured by electromyography (EMG)) was more active in musicians and was interpreted as a (perhaps voluntary) dislike reaction of musicians to the experimental stimuli [159].

More recently, Park and colleagues [160] investigated the relation between musical expertise and neural correlates of happiness, sadness and fear in music by means of fMRI, obtaining both higher arousal ratings and increased activations in the right dorsolateral prefrontal cortex and in right parietal regions in response to sad and fearful music, respectively. Happy music instead did not activate differentially the brain of musicians and non-musicians. A recent study by Alluri *et al.* [161] further investigated the functional connectivity between the nucleus accumbens and the rest of the brain during music listening and how it is affected by musical expertise. The findings indicate that the left nucleus accumbens has increased connections with other regions of the limbic and paralimbic system in musicians, whereas the right nucleus accumbens becomes more coupled to the hippocampus in non-musicians and to the temporal pole and ventromedial frontal regions in musicians. These results confirm and extend the findings of increased connectivity comprising the nucleus accumbens and the frontal regions during rewarding music listening, obtained by [162]. These results are novel in showing that the connectivity of the regions controlling reward and pleasurable experience with regions related to motor control and cognitive processing increases with musical expertise.

Overall, the above reviewed results showing the stronger reactivity of the central and autonomous nervous system in musicians during the affective processing of music contrast the former conception of musicians as having a cognitive, detached approach when listening to music [163,164], suggesting instead a more intense, perhaps more embodied and proprioceptive experience during music listening in musicians as compared with non-musicians [134].

Supporting the previous interpretation, previous authors obtained increased anterior insula activity in musicians as opposed to non-musicians [165]. The insula is a brain structure, hidden deeply inside the Sylvian fissure, which contains a map of the visceral sensations coming from the body, in its middle part. The anterior part of the insula is hypothesized to integrate those visceral sensations into a conscious percept, which results in proprioception and awareness of one’s own body place in the world [166]. Proprioception is indeed very relevant during musical practice and in prolonged musical experiences (whether active, during performance or practicing, or passive, during listening). For instance, beginner musicians continuously control in a conscious way the position and movements of their bodies, whereas experts do not [167]. However, experts need what Gallagher [168] calls “performative awareness” of the body, even while not paying attention to specific parts: “With proprioception as a basis, we recognize that our feelings, movements, thoughts, and beliefs are indeed our own” [167] (p. 220). The deeper awareness of one’s own body in musicians and particularly in classically trained instrumentalists might be at the origins of the findings by Zamorano *et al.* [169], who reported their superior tactile sensitivity, corresponding to cortical reorganization of somatosensory cortices. In more detail, the tactile thresholds (obtained with three distinct paradigms, namely mechanical detection, grating orientation and two-point discrimination) were lower, and the subjective pain ratings to thermal and pressure stimuli were higher in musicians, healthy or with chronic pain, and in non-musicians suffering from chronic pain, as opposed to healthy non-musicians, indicating lower tactile spatial acuity and increased pain sensitivity depending on pain and musical expertise. The authors of the study link the findings to previous observation of experience-dependent maladaptive changes in the receptive fields of the somatosensory cortices following intensive training of repetitive, highly skilled movements of the limbs and to the higher risk of developing chronic pain in classical instrumental musicians [170].

To summarize, research on the modulating role of musical expertise on emotional processing of music in the brain is only at its infancy. Yet, some claims are almost conclusive even at this stage of research: music is a powerful tool for emotion and mood modulation by triggering ancient evolutionary systems, such as the limbic system, the brain stem and related structures [171], and experience with playing a musical instrument might shape the brain mechanisms guiding how emotions are perceived and induced by music and even how they are felt proprioceptively in the body.

## 6. Evaluation and Preference

The evaluative aspects of a musical experience are rarely studied in music neuroscience. However, a very typical and almost unavoidable reaction to music is that which leads us to decide if we like or not a musical piece. Aesthetic judgments of musical beauty have been studied with respect to musical expertise in an ERP study by Müller *et al.* [172]. They measured musicians while they listened to two-second long sequences, containing five chords, and they judged in the first (randomly shuffled) listening whether they sounded correct or incorrect and in another listening whether they sounded beautiful or not. The sequences followed only partially the rules of Western tonal music, allowing for some ambiguity and variation of judgment ratings. The results showed that stimuli rated as beautiful evoked a prominent brain response previously associated with late affective processing (the late positive potential) only in non-musicians, suggesting that they relied on affect processes and induction to issue an aesthetic evaluation, whereas musicians utilized more cognitive strategies.

Likely, also the desire to comply with *social expectations* might affect the evaluative judgments of music, as tentatively shown by Berns *et al.* [173] with fMRI. They found that adolescents changed their ratings of heard song excerpts and also their brain responses, after becoming aware of the popularity ratings of the same songs by other peers who participated in the study.

Appreciation of music, therefore, is complex and is dependent on sociocultural factors, experience and memory, suggesting “an intricate interplay between the dopaminergic system and cortical regions that contain previously acquired sound templates, track temporal and hierarchical structure, integrate emotions with reward value, detect internal states, assign reward value to stimuli, and make value-based decisions about reward-related stimuli” [8].

Apart from the ones described above, thus far, we are not aware of other studies investigating the question of whether musical expertise can alter the neural mechanisms for the liking or beauty evaluation of music. This important aspect of the aesthetic musical experience, which leads to the decision to listen to music and purchase a musical product, remains to be further elucidated.

## 7. Concluding Remarks

In this contribution, we have adopted and updated a comprehensive framework of a musical aesthetic experience, which incorporates several subprocesses ordered chronologically and covering perceptual, cognitive, affective, proprioceptive, evaluative and social functions, as well as the subjectivity of the music agent (see Figure 2). This framework benefits from a cybernetic and ecological view of “organism-environment interactions”, forcing “immediate” internal changes (see Figure 1) in a continuous attempt to make sense of the perceptual flux. As such, we read the literature on music-derived neuroplasticity, structuring it on the basis of the subprocesses comprising a musical aesthetic experience. By doing this, we stressed the need for further research on important aspects of music playing and listening, such as proprioception, aesthetic evaluation and social interaction.

The cybernetics approach further evidences the “immediate” alterations of the internal state and even the structure of the music agent due to the continuous adjustments of his or her sensor and effector organs in order to adequately respond to the dynamic dispositions of the environment. Most of the reviewed studies utilize conventional paradigms in which one or a few features (whether acoustic or emotional or cognitive) are manipulated in order to elicit a brain or behavioral response to them. The “evoked response technique” (whether recorded with EEG or magnetoencephalography, MEG; in the first case referred to as ERP, in the latter as event-related field, ERF) allows the recording of immediate reactions to the feature within one second after the feature onset, but in its most common paradigm, it mostly has the drawback of requiring tens to hundreds of repetitions of the same feature parameter before the brain signal can be extracted from the noise baseline (the continuous neural activity combined with the measurement errors). Recordings using this technique thus employ stimulation consisting of isolated sounds or sound patterns. The adoption of realistic music, e.g., from commercial albums, having some ecological validity, can instead be measured with fMRI. This measurement technique, unfortunately, has lower temporal resolution than the evoked response technique, being around two seconds or more; this means that only an averaged account of the neural signal to musical sounds is obtained, missing the fast adaptations to the continuous real-time changing acoustic features. The often used sparse sampling design of fMRI, furthermore, involve short clips of musical pieces in order to allow for the noisy and potentially interfering volume acquisition to occur in the interval between the stimuli or else to allow for the behavioral responses of the subjects [174,175,176,177].

In order to study the dynamics of these “immediate” changes of internal state and structure during music listening, a new paradigm has been recently introduced by Alluri *et al.* [146]. In this paradigm, the subjects listen continuously to whole musical pieces, and the study of the dynamic brain responses to musical features is made possible by correlating the brain signal to the acoustic features that have been computationally extracted from the music (by means of the MIRToolbox of MATLAB). In their study, Alluri *et al.* extracted 25 features and reduced their dimensionality by means of principal component analysis (PCA), as well as by means of a listening test, obtaining six acoustic components that were correlated subsequently with the time series of the continuous brain signal measured with fMRI. Future investigations could employ the above-described approach and analyze how the “immediate” dynamic reactions to music alter the internal state of the “music agent”.

To conclude, the neural adaptations reviewed here, and conceived of as the consequence of lifelong interactions with musical multisensorial stimuli, are observed in all of the subprocesses comprising a musical aesthetic experience, towards the final goal of achieving a better perceptual, motor and proprioceptive response to the immediate demands of the sounding environment. The present review, however, evidenced also the gaps in research concerning some of these subprocesses. New studies are called for to obtain detailed knowledge on how the resulting neural adaptations are dependent on the specific content of the musical interactions (attentional involvement, aesthetic stance, amount of musical training, starting age, musical genre played) and particularly on the motor and/or proprioceptive components of these interactions.
